# Glatiramer Acetate modulates ion channels expression and calcium homeostasis in B cell of patients with relapsing-remitting multiple sclerosis

**DOI:** 10.1038/s41598-018-38152-8

**Published:** 2019-03-12

**Authors:** C. Criscuolo, A. Cianflone, R. Lanzillo, D. Carrella, A. Carissimo, F. Napolitano, R. de Cegli, P. de Candia, C. La Rocca, T. Petrozziello, G. Matarese, F. Boscia, A. Secondo, D. Di Bernardo, V. Brescia Morra

**Affiliations:** 1 Department of Neurosciences, Reproductive and Odontostomatological Sciences, “Federico II” University of Naples, 80131 Naples, Italy; 2 IRCCS SDN, Napoli, Italy; 3 Telethon Institute of Genetics and Medicine (TIGEM), Pozzuoli, (NA) 80078 Italy; 4 Institute for Applied Mathematics ‘Mauro Picone’, National Research Council, Naples, Italy; 5 IRCCS MultiMedica, 20138 Milan, Italy; 6 Istituto per l’Endocrinologia e l’Oncologia Sperimentale, Consiglio Nazionale delle Ricerche (IEOS-CNR), 80131 Napoli, Italy; 7 Dipartimento di Medicina Molecolare e Biotecnologie Mediche, Università di Napoli Federico II, 80131 Napoli, Italy; 8 Division of Pharmacology, Department of Neuroscience, Reproductive and Odontostomatological Sciences, School of Medicine, “Federico II” University of Naples, Naples, Italy; 9 Department of Chemical, Materials and Industrial Production Engineering, University of Naples Federico II, 80125 Naples, Italy

## Abstract

To investigate the effects of Glatiramer Acetate (GA) on B cells by an integrated computational and experimental approach. GA is an immunomodulatory drug approved for the treatment of multiple sclerosis (MS). GA effect on B cells is yet to be fully elucidated. We compared transcriptional profiles of B cells from treatment-naïve relapsing remitting MS patients, treated or not with GA for 6 hours *in vitro*, and of B cells before and after six months of GA administration *in vivo*. Microarrays were analyzed with two different computational approaches, one for functional analysis of pathways (Gene Set Enrichment Analysis) and one for the identification of new drug targets (Mode-of-action by Network Analysis). GA modulates the expression of genes involved in immune response and apoptosis. A differential expression of genes encoding ion channels, mostly regulating Ca^2+^ homeostasis in endoplasmic reticulum (ER) was also observed. Microfluorimetric analysis confirmed this finding, showing a specific GA effect on ER Ca^2+^ concentration. Our findings unveils a GA regulatory effect on the immune response by influencing B cell phenotype and function. In particular, our results highlight a new functional role for GA in modulating Ca^2+^ homeostasis in these cells.

## Introduction

Traditionally, multiple sclerosis (MS) has been considered to be a CD4^+^ T mediated disorder but recent evidences have challenged this idea by indicating a role also for B lymphocytes in the pathogenesis of MS^1–3^.

Not surprisingly B lymphocytes have become a target of novel pharmacotherapies in MS (i.e. ocrelizumab). However, many MS existing disease-modifying therapies (DMTs), beside their specific mechanism of actions, have the potential to affect B cell behaviour as well. Glatiramer acetate (GA) is a polypeptide-based therapy approved for the treatment of relapsing remitting (RR)-MS^4^. The mode of action of GA is only partially known, and specifically its effect on B cells is not completely understood. Data from literature show that GA treatment of MS patients decreases CD19+ B cells and memory B cells and increases peripheral blood naïve B cells leading to a less disease promoting B cell phenotype^5^. On the other hand, GA affects B cells functions, inducing B cell expression of anti-inflammatory cytokines and inhibiting pro-inflammatory cytokines^6^. Elucidating the mechanisms of action of drugs in clinical use for MS is crucial for a concrete improvement of MS pathogenesis knowledge. Therefore, we decided to investigate GA effects, at the transcriptional level, on treatment-naïve RR-MS patients’ B cells by using an integrated computational and experimental approach. Indeed, it has been shown that transcriptional responses of cells treated with small molecules can be used to elucidate their mechanism of action, in the lead optimization phase of drug discovery project and to reveal similarities among drugs, and quickly transfer indications for drug repositioning^7,8^. The Connectivity Map (CMAP), the largest peer-reviewed public database of gene expression profiles following treatment of five human cancer cell lines with 1309 different bioactive small molecules has been extensively used by both the academic and industrial communities^9^.

We hypothesized that measuring the transcriptional response on patients’ B cells following drug treatment and comparing this response to those of drugs collected in the CMAP database would help revealing the mechanism of action of GA in these cells. To this end, we applied a bioinformatics tool named Mode of Action by NetwoRk Analysis (MANTRA) (http://mantra.tigem.it) that was recently described in the literature^7,8^. MANTRA is an automatic and robust approach that assesses similarity in gene expression profiles following drug treatment across thousands of small molecules from the CMAP database to predict similarities in drug effect and Mode of Action (MoA). Briefly, each transcriptional response to a drug treatment is represented as a list of genes ranked according to how much they change with respect to untreated cells. Transcriptional similarity among two drugs (i.e. two ranked lists of genes) is quantified by assessing how much two drugs tend to upregulate and downregulate the same genes. MANTRA represents transcriptional similarities among drugs graphically in the form of a network, where two drugs are connected if they are transcriptionally similar according to a metric based on the Gene Set Enrichment Analysis^7^. The position of a drug within the network provides insights about its MoA by exploiting previous knowledge on neighbouring drugs^7,8^. In addition, the network can be partitioned into communities consisting of groups of densely interconnected drugs sharing similar mode of action. The CMAP database is thus represented in MANTRA as a network of 1,309 drugs grouped into 106 communities^7^. MANTRA has been shown to be able to predict drug mechanism of action and functional targets from the analysis of gene expression profiles and for drug repositioning^10^.

Here, by performing a bioinformatics analysis of the CMAP dataset together with gene expression profiles of B cells from patients treated with GA, we revealed a possible role of this drug in modulating immune response, apoptosis, and ion channels expression, in particular voltage gated- and store-operated Ca^2+^ channels. To support these findings, we investigated endoplasmic reticulum (ER) Ca^2+^ content in B cells from MS patients treated with GA vs healthy subjects, and vs drugs-naïve RR-MS patients or treated with other first line DMTs.

## Results

### Generation of gene expression profiles

**Figure 1:**
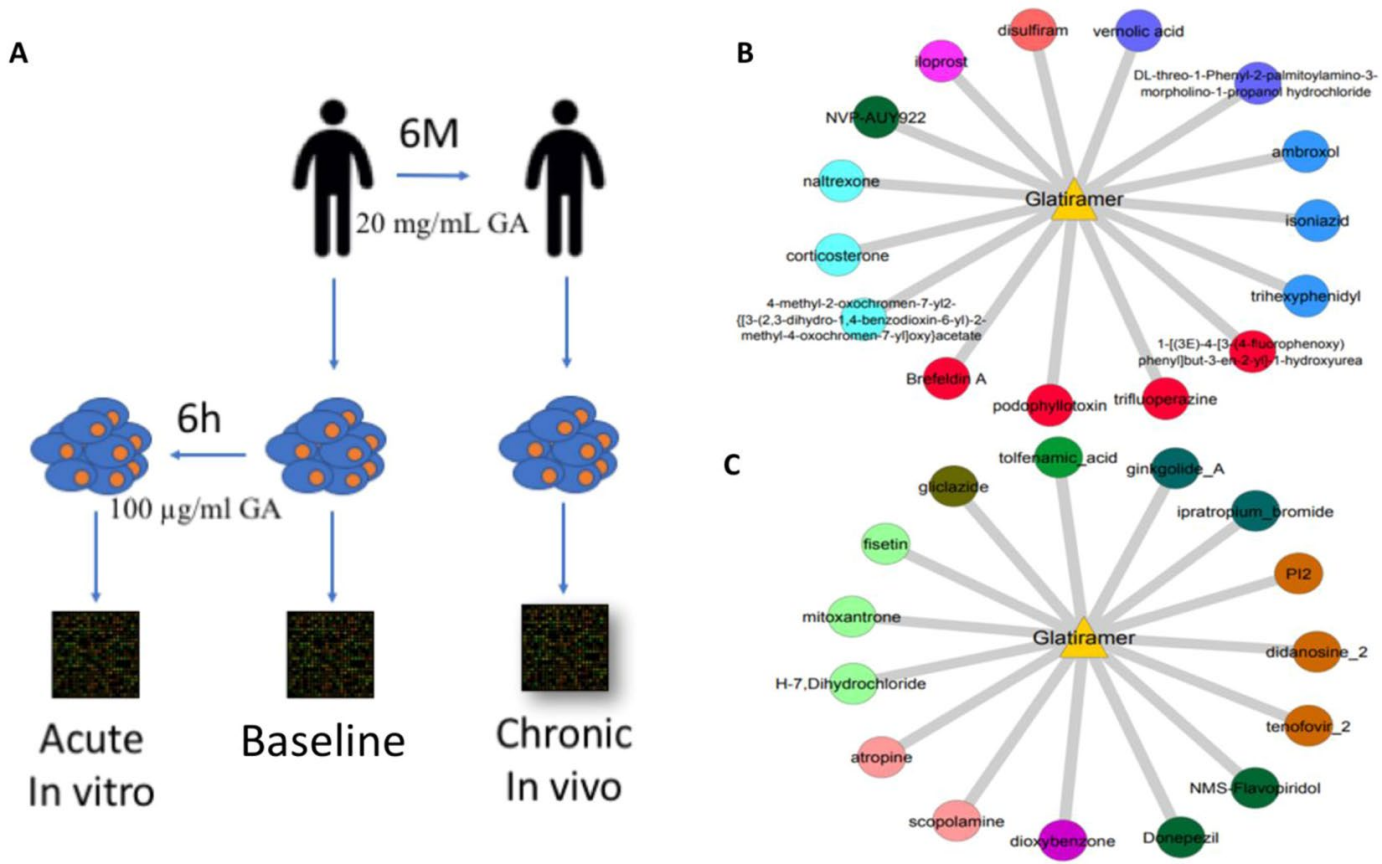
MANTRA analysis of gene expression profiles obtained from patients’ B cells following acute *in vitro* or chronic *in vivo* treatment with glatiramer acetate. (**A**) Schematics of experiment. (**B**,**C**) MANTRA analysis. The triangle represents glatiramer acetate (GA) and edges connecting GA to circles represent drugs inducing a similar transcriptional profile according to the Connectivity Map. The top 15 most similar drugs are shown for the acute *in vitro* (**B**), and for the chronic *in vivo* treatment (**C**). Compounds distances from GA were equal or less than 0.8. Node colors indicate communities.

Ten consecutive treatment-naïve patients with RR-MS were enrolled between July 2013 and March 2014. One patient was successively excluded from the study for pregnancy. All patients were females. Mean age at enrolment ± SD was 31.5 ± 2.07. Mean age at MS onset was 29.16 ± 4. Mean EDSS ± SD was 2.5 ± 0.5. Specifically, as shown in Fig. 1A,B cells of each patient were isolated at baseline (treatment free cells, before starting GA) and after six months of 20 mg/mL GA *in vivo* administration (*in vivo* treated cells). At baseline, B cells were also cultured and treated or not *in vitro* with 100 µg/ml GA for six hours Fig. 1A. Acute (6 hours, *in vitro*) and chronic (6 months, *in vivo*) responses to GA treatment in B cells were then analyzed at the transcriptional level by Affymetrix microarrays (GSE110023). RNA quality of three patients was poor therefore microarray data were analyzed only in 6 patients (60%).

### Functional pathway and MANTRA analysis of acute (*in vitro)* transcriptional response to GA treatment

Gene expression profiles of B cells following 6 hour GA treatment *in vitro* were compared to those of untreated cells in order to detect changes in gene expression caused by the drug treatment across the 6 patients. Genes were ordered according to their fold-change in treated versus untreated samples. To assess the molecular pathways modulated *in vitro* by GA, we performed Gene Set Enrichment Analysis (GSEA). GSEA uses one or more databases of set of genes (i.e. pathways) to identify those gene-sets which are significantly modulated by the treatment. In this study, we selected as pathway databases those of gene expression signatures of immune system cells (C7) and of Gene Ontology (GO) including biological processes, cellular components and molecular function (C5). Pathway enrichments were evaluated by their normalized enrichment score (NES), nominal *P* value, and FDR. The most significant GO pathways identified by GSEA included those involved in translation, which were up-regulated, and those involved in ion channels expression, including calcium, which were down-regulated (Table 1). GSEA of immune system signatures highlighted up-regulation of genes specific to naïve T and B cells (Table 1).

**Table 1:** Significant gene sets down-regulated and up-regulated obtained by GSEA performed after *in vitro* glatiramer acetate treatment (acute response) and after *in vivo* six months glatiramer acetate administration to patients (chronic response).

MSigDB collections	Sub-collections	Gene Set name	SIZE	NES	NOM p-val	FDR q-val
**Acute response**						
C5:	MF	STRUCTURAL_CONSTITUENT_OF_RIBOSOME	71	1,7717452	0	0,07397725
C5:	MF	VOLTAGE_GATED_POTASSIUM_CHANNEL_ACTIVITY	32	−2,7496712	0	0
C5:	MF	LIGAND_GATED_CHANNEL_ACTIVITY	36	−2,3845527	0	0
C5:	MF	VOLTAGE_GATED_CALCIUM_CHANNEL_ACTIVITY	18	−2,3448026	0	0
C5:	MF	VOLTAGE_GATED_CATION_CHANNEL_ACTIVITY	59	−3,215854	0	0
C5:	MF	CALCIUM_CHANNEL_ACTIVITY	32	−1,4731557	0.0	0,04459786
C7:	immunologic signatures	NAIVE_BCELL_VS_NEUTROPHIL_UP	175	1,6156584	0.0	0,001
C7:	immunologic signatures	NAIVE_BCELL_VS_BM_PLASMA_CELL_UP	174	1,4851125	0.0	0.00728678
C7:	immunologic signatures	NAIVE_VS_MEMORY_CD8_TCELL_UP	129	1,4530996	0.001	0.011323772
C7:	immunologic signatures	NAIVE_TCELL_VS_NEUTROPHIL_UP	168	1,4417284	0.0	0.012546144
C7:	immunologic signatures	BCELL_VS_MONOCYTE_UP	165	1,5381814	0.0	0.0028463998
**Cronic response**						
C5:	MF	GATED_CHANNEL_ACTIVITY	108	2,9445336	0	0,00108302
C5:	MF	CATION_CHANNEL_ACTIVITY	106	2,849798	0	0,00180962
C5:	MF	SUBSTRATE_SPECIFIC_CHANNEL_ACTIVITY	135	2,832006	0	0,06168386
C5:	MF	METAL_ION_TRANSMEMBRANE_TRANSPORTER_ACTIVITY	132	2,5542498	0	0,02961846
C5:	MF	VOLTAGE_GATED_CALCIUM_CHANNEL_ACTIVITY	18	2,4818778	0,00180505	0,05882143
C5:	MF	VOLTAGE_GATED_POTASSIUM_CHANNEL_ACTIVITY	32	1,8135619	0,01344538	0
C5:	BP	SODIUM_ION_TRANSPORT	15	2,1463487	0,00367647	0,00396349
C5:	BP	POTASSIUM_ION_TRANSPORT	51	1,9955596	0,00172712	0,01392227
C5:	MF	TRANSMEMBRANE_RECEPTOR_PROTEIN_TYROSINE_KINASE_ACTIVITY	43	2,7663453	0	0,01309908
C5:	MF	AMINE_BINDING	20	2,3429487	0	0,01807819
C5:	BP	NERVOUS_SYSTEM_DEVELOPMENT	314	2,4207823	0	0,02556699
C5:	BP	NEUROLOGICAL_SYSTEM_PROCESS	324	2,3578296	0	0,01907202
C5:	BP	SYNAPTIC_TRANSMISSION	152	2,2413225	0	0,0320724
C5:	BP	TRANSMISSION_OF_NERVE_IMPULSE	165	2,1886022	0,00176991	0,03053605
C5:	BP	CELLULAR_BIOSYNTHETIC_PROCESS	259	−2,3858764	0	0,02961543
C5:	BP	NEGATIVE_REGULATION_OF_PROGRAMMED_CELL_DEATH	131	−2,1043394	0	0,02761854
C5:	BP	CELL_STRUCTURE_DISASSEMBLY_DURING_APOPTOSIS	16	−2,103058	0,00217391	0,02629634
C5:	BP	NEGATIVE_REGULATION_OF_APOPTOSIS	130	−2,0976427	0	0,01524475
C5:	BP	POSITIVE_REGULATION_OF_IMMUNE_RESPONSE	22	−2,0900369	0,002331	0,02761854
C5:	MF	INFLAMMATORY_RESPONSE	107	−2,0895972	0	0,02629634
C7:	immunologic signatures	CHEMOKINE_ACTIVITY	37	−2,2534335	0,00228833	0,01524475
C7:	immunologic signatures	IGM_VS_SWITCHED_MEMORY_BCELL_UP	175	2,538935	0	0
C7:	immunologic signatures	IGM_MEMORY_BCELL_VS_PLASMA_CELL_DN	176	1,8842129	0,002	0,017
C7:	immunologic signatures	NAIVE_VS_SWITCHED_MEMORY_BCELL_UP	166	2,1028352	0	0,004
C7:	immunologic signatures	NAIVE_BCELL_VS_NEUTROPHIL_UP	175	−3,4884412	0.0	0.0
C7:	immunologic signatures	NAIVE_TCELL_VS_NEUTROPHIL_UP	168	−3,3898792	0.0	0.0
C7:	immunologic signatures	NAIVE_TCELL_VS_DC_UP	173	−3,1822155	0.0	0.0
C7:	immunologic signatures	BCELL_VS_MDC_UP	162	−2,8965137	0.0	0.0
C7:	immunologic signatures	BCELL_VS_MONOCYTE_UP	165	−2,749503	0.0	0.0

We then analysed the ranked list of genes following acute treatment of B cells with GA to identify drugs inducing a significantly similar transcriptional response, and hence which could share a common mechanism of action with GA by means of the MANTRA online tool^7,8^. MANTRA identified 42 drugs eliciting a significantly similar transcriptional response to the *in vitro* GA treatment (Fig. 1B and in Supplementary Table S1). The 42 drugs were part of 17 communities sharing similar mode of action^7^. Interestingly, among the 42 drugs we found agents with antiinfiammatory effects (estradiol, corticosterone etc.), widely used for the treatment of relapses in MS and drugs such as vigabatrin an antiepilectic able to suppress voltage-sensitive sodium channels^11^, and naltrexone, an opioid antagonist proposed against spasticity, pain and fatigue in MS^12^, which has been shown to prevent relapses in MS and to reduce the disease^13^.

### Functional pathway and MANTRA analysis of chronic (i*n vivo)* transcriptional response to GA treatment

Gene expression profiles of patients’ B cells after six months of GA administration (*in vivo)* were compared to untreated cells of each patient at baseline (before starting GA treatment). We again performed GSEA and found a significant up-regulation of genes encoding ion channels (Table 1), such as genes belonging to the calcium voltage-gated channel subunit (CACN) and to the transient receptor potential (TRP) channel (TRPC1-5, TRM2, TRPV2) families. TRPC1, TRPC3 and TRPC4 are molecular component of store-operated Ca^2+^ entry (SOCE), an ubiquitous Ca^2+^ entry pathway that is activated in response to depletion of ER-Ca^2+^ stores^14,15^. Ca^2+^ signal, initiated by Ca^2+^ release from ER is evoked by tyrosine kinase receptors phosphatase C activation^16^. Interestingly, GSEA shows also the induction of genes involved in protein tyrosine kinase activity, amine-binding function and in nervous system development and function.

A general down-regulation of pathways involved in negative regulation of cell death and cellular biosynthetic process was also identified by GSEA (Table 1). As in the case of acute response, GSEA for pathways related to the immune system identified a significant up-regulation of genes linked to a less mature B cell phenotype. On the contrary, genes specific for lymphocytes vs others cells were down-regulated (naïve _bcell_vs_neutrophil_up, bcell_vs_monocyte_up), while in the acute response these were up-regulated.

We then performed MANTRA analysis of the B cells *in vivo* transcriptional response to GA. MANTRA identified a total of 49 drugs grouped into 18 communities (Fig. 1C and Supplementary Table S1). The communities with the largest number of drugs included community n. 14 (cyclin-dependent kinases 2 (CDK2) and Topoisomerase II inhibitors) with 6 drugs, community n. 89 (hemostatic agents) with 7 drugs, and community 90 (mainly anti-inflammatory, antibacterial and antiretroviral compounds) with 14 drugs.

### Effect of GA on [Ca^2+^]_i_ homeostasis in B cells

**Figure 2:**
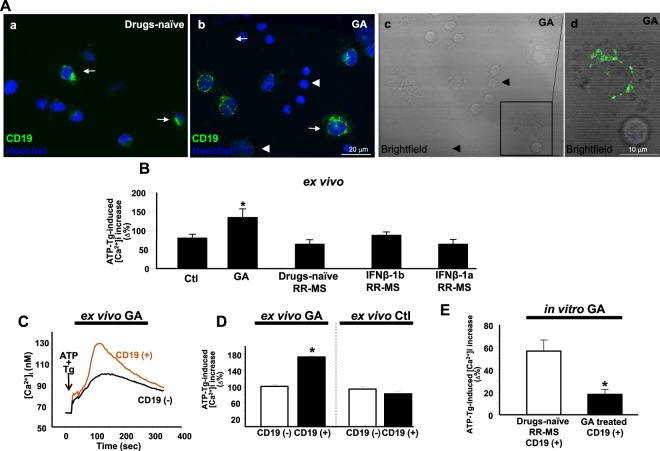
Effect of glatiramer acetate on [Ca^2+^]_i_ homeostasis. (**A**) Confocal immunofluorescence images depicting FITC-CD19^+^ B cells in PBMCs isolated from naïve (a) or glatiramer acetate (GA) (b) patients. Panel c shows the brightfield image of panel b; d, higher magnification of the frame in c showing a single FITC-CD19^+^ cell. Arrows point to FITC-CD19^+^ cells, arrowheads point to FITC-CD19^−^ cells. (**B**) Quantification of *ex vivo* microfluorimetric experiments showing the effect of ATP+ thapsigargin on [Ca^2+^]_i_ in PBMCs from healthy subjects (Ctl) (N = 80 cells), from patients treated with GA (N = 40 cells), interferon (INF) β-1b (N = 40 cells), INFβ-1a (N = 40 cells), and from drugs-naïve patients (N = 50 cells). *p < 0.05 vs all. (**C**) Representative traces of the effects of ATP+ thapsigargin on [Ca^2+^]i in FITC-CD19^+^ and FITC-CD19^−^ cells of PBMCs isolated from GA treated patients. (**D**) Quantification of the effects of ATP+ thapsigargin on [Ca^2+^]i in FITC-CD19^+^ and FITC-CD19^−^ cells from GA treated patients and healthy subjects. *p < 0.05 vs all. (**E**) Quantification of the effects of ATP+ thapsigargin on [Ca^2+^]i in FITC-CD19^+^ cells obtained from drugs naïve RR-MS patients treated or not *in vitro* with GA. *p < 0.05 vs drug-naïve RR-MS. Data are expressed as mean ± S.E.M of three independent experiments for each condition. Statistical analysis was performed using the one-way ANOVA or t-test followed by Newman Keul’s test. p < 0.05 was considered statistically significant.

In order to better investigate the computational results hinting at a role of GA in modulating Ca^2+^ channels expression, we analyzed the effect of GA treatment on ER [Ca^2+^]_i_ homeostasis. We recorded PBMCs form three healthy subjects and from 13 RR-MS patients: three disease-treatment free, four treated with GA, six with INFβ-1a and 1b (Table 2). Confocal immunofluorescence analysis showed that the anti-CD19 antibodies clearly recognized B cells both in PBMCs isolated from healthy subjects and GA-treated patients (Fig. 2A,a,b). In this latter group, CD19^+^ cells appeared surrounded by several vesicles when observed in brightfield microscopy. (Fig. 2A,c,d).

**Table 2:** Demographic and clinical characteristics of patients and controls enrolled for Ca^2+^ homeostasis study.

	Controls	GA RR-MS patients	INF-β1b RR-MS patients	INF-β1a RR-MS patients	Drug naïve RR-MS patients
Total	3	4	3	3	3
Age (years; mean ± SD)	30 ± 7.0	33.25 ± 5.4	34.5 ± 1.1	35.5 ± 3.5	31.5 ± 3.5
Age at onset (years; mean ± SD)		30.5 ± 7.4	26.5 ± 3.5	28 ± 6.5	
DD (years; mean ± SD)		8.75 ± 5.8	11.5 ± 3.7	9.5 ± 4.0	
EDSS (mean ± SD)		2.375 ± 1.0	2.25 ± 0.3	3.225 ± 0.2	2.5 ± 0

Microfluorimetric experiments showed that the ER Ca^2+^ content was significantly higher in PBMC isolated from GA-treated patients than in those isolated from healthy subjects, disease-treatment free, or INFβ1a and 1b treated patients (p = 0.02; Fig. 2B). To assess whether the observed changes in Ca^2+^ content occurred in B cells, we recorded the ER Ca^2+^ content in both FITC-CD19^+^ and FITC-CD19^−^ cells. Interestingly, microfluorimetric analysis revealed that the ER Ca^2+^ release elicited by ATP+ thapsigargin was significantly higher in FITC-CD19^+^ cells than in FITC-CD19^−^ cells from patients treated with GA (p = 0.01), whereas the ER Ca^2+^ content was similar in FITC-CD19^+^ and FITC-CD19^−^ cells obtained from healthy subjects (Fig. 2C,D).

Conversely, when FITC-CD19^+^ cells from disease-treatment free RRMS patients were exposed *in vitro* to 100 µg/ml GA for 8/10 hours, the ER Ca^2+^ content was significantly lower than in untreated cells (p = 0.008; Fig. 2E).

## Discussion

Our functional analyses of transcriptional profiles after both acute and chronic GA treatment revealed two main perturbated biological functions: (1) immune response and (2) ion channel activity.

The first GA action on immune response seems to be on B phenotype switch from a more to a less mature one. Indeed the acute response shows a relative decrease in plasma cells or memory B cells with concomitant expansion of B-cell precursors and/or naïve B cells. These data are in line with the literature^5,6^. Given the short time of GA exposure for acute treatment, we may only appreciate the initiation of the cells phenotypic switch phenomenon.

In chronic response, we were able to confirm that GA targets immune response by: (1) affecting B cells phenotype, (2) affecting B cells function (down-regulation of inflammatory pathways), (3) reducing the number of lymphocytes (down-regulation of lymphocytes vs other blood cells pathways).

The apoptosis induction, revealed by GSEA analysis, may be consequence of GA modulation on immune system rather than a direct effect.

Ion channels genes are down-regulated in acute treatment (*in vitro*) but up-regulated in chronic GA treatment (*in vivo*).

A possible explanation of this opposite response to GA may be a compensatory effect where GA treatment acutely down-regulates ion channels genes in B cells, which in turn causes a chronic upregulation of these genes to maintain homeostasis.

Ion channels genes, whose expression changes following GA treatment, belong the repertoire of ion-conducting proteins like potassium and chloride, Ca^2+^ release-activated Ca^2+^ (CRAC) and TRP channels. These last channels participate to ER Ca^2+^ refilling^17,18^ which represents the major Ca^2+^ influx mechanism in B lymphocytes controlling antigen-mediated lymphocyte activation, cytokine/chemokine production, exocytosis, enzyme control, proliferation and apoptosis^19–21^. In line with the bioinformatic findings, intracellular Ca^2+^ concentration was reduced *in vitro* by GA but increased *ex vivo*.

A role of GA on intracellular Ca^2+^ has been already reported in platelets where, similar to our findings, intracellular Ca^2+^ was reduced after 100 µg of GA *in vitro*^22^. However, if ion channels expression represents a trigger or a consequence of Ca^2+^ homeostasis modifications is still to be elucidated.

After acute treatment, according to MANTRA, Brefeldin-A (BFA) and Ambroxol had the most similar gene expression profile to GA. BFA is an inhibitor of intracellular protein transport that leads to blockade the forward transport between the ER and the Golgi apparatus^23^. When anterograde transport is suppressed, retrograde transport from the Golgi to the ER becomes apparent. This BFA-induced retrograde transport depends on Ca^2+^ sequestered into intracellular stores^24^. Therefore, it is possible that, due to GA modulation on Ca^2+^ homeostasis, and through second messengers and transcription factors, the mode of action of the two drugs could be similar at transcriptional level.

Ambroxol is a mucolytic agent acting by inhibiting Na^+^ channel and by reducing nitric oxide levels^25^. Ambroxol has also antiinflammatory properties including inhibition of oxidative stress, increased of local defense and reduction of pro-inflammatory cytokines^26^. By blocking voltage gated Na+ channels and Ca^2+^ channels in animal models Ambroxol promotes axon regeneration in the injured adult mouse central nervous system^27^.

The compounds most transcriptionally similar to chronic GA treatment belonged to the Topoisomerase II and CDK2 inhibitors drug class, agents widely used for human cancer treatment that prevent unregulated cancer cell proliferation leading to apoptosis. Considering these proapoptotic drugs, MANTRA results are in line with GSEA analysis *in vivo*, that showed a downregulation of pathways involved in negative regulation of apoptosis (Table 1). Among these compounds, MANTRA identified Mitoxantrone (Fig. 1C), currently used for the treatment of secondary progressive or worsening RR-MS^28^. This is noteworthy, since it proves that our protocol was able to identify a previously proven drug against MS.

In conclusion, our combined computational and experimental study hints at a possible role of GA in B cells as a modulator of Ca^2+^ homeostasis. We suggest that GA interferes with B cell activity by tuning ion channels expressions and Ca^2+^ influx, crucial for the regulation of B cell differentiation, cytokine production and apoptosis.

## Methods

### Subjects

Patients were recruited at MS center of the Department of Neurosciences, Reproductive and Odontostomatological Sciences of Federico II University of Naples. Patients’ inclusion criteria were: (1) new diagnosis of RR-MS according to Mc Donald revised criteria^29^; (2) presence of oligoclonal bands in the cerebrospinal fluid; (3) DMTs naïve; (5) age between 30 and 35 years; (4) patients should have not been treated with corticosteroid for at least one month at enrolment. Written informed consent was obtained from each patient. All methods were performed in accordance with the relevant guidelines and regulations and all experimental protocols were approved by the local ethical review committee of the University of Naples Federico II. Patients underwent GA treatment 20 mg daily s.c. as for clinical practice, and followed up for six months.

In a second phase, to ascertain computational results, we enrolled new healthy controls and patients treated with GA, with INFβ-1a and 1b, and treatment naïve.

### B cells isolation and treatment

Patients’ peripheral blood samples were collected at baseline and after six months of GA *in vivo* administration.

Peripheral blood mononuclear cells (PBMC) were separated using Ficoll-Paque centrifugation (GE Healthcare); B cells were purified by autoMACS Pro Separator (Miltenyi biotec) using B cell Isolation kit II (Miltenyi Biotec, Bergisch Gladbach, Germany). The isolated cells, were analyzed using a FACS CANTO II (BD Biosciences, CA, USA) to confirm the purity of B cells (>98%). Cells at baseline were divided in two group. One underwent RNA extraction like B cells successively isolated from the same  MS patient after six months of GA. The second group was seeded (1 × 10^6^ cells/ml) with RPMI-1640 medium supplemented with 100 UI/ml penicillin, 100 μg/ml streptomycin (Life Technologies) and 5% autologous serum. Cultured cells were treated *in vitro* or not with 100 μg/ml GA for six hours in a humidified atmosphere containing 5% CO^2^ at 37 C°. All experiments were carried out in duplicate.

### Microarray experiments

Microarray hybridization experiments on total RNA extracted with TRIzol reagent (Ambion), were performed at Coriell Insitute for Medical Research, Camden, NJ. The Affymetrix Gene-Chip Human Genome HG-U133A_2 hybridization platform (Affymetrix, Santa Clara, CA) was performed as described previously^30^. Prior to hybridization, RNA was reverted to cDNA using the NuGen Ovation RNA-Seq System V2 (NuGen Technologies, San Carlos, CA).

### Bioinformatics analysis

#### Microarray analysis: GSEA and MANTRA

Microarray data were pre-processed using the Bioconductor package Affy and normalized with the RMA method^31^. Differentially expressed genes (DEGs) between conditions (GA-treated versus untreated B cells *in vitro* and *in vivo*) were identified using a Bayesian *t*-test^32^. For each *P* value, the Benjamini–Hochberg procedure was used to calculate the false discovery rate (FDR) to correct for multiple testing.

The Microarray data have been deposited in NCBIs Gene Expression Omnibus (GEO) and are accessible through GEOSeries accession number GSE110023^33^.

Gene set enrichment analysis (GSEA) was then performed using the freely available software GSEA v2.0 from the Broad Institute^34^. To run the GSEA algorithm, RMA-normalized microarray data were used. The GSEA algorithm collapsed the probe sets into gene symbols (~13,300 genes) and ranked the genes on the basis of the fold change after GA treatment (*in vitro* or *in vivo*). An enrichment score was calculated for each gene-set by applying the Kolmogorov-Smirnov statistics as described in the GSEA algorithm. Gene-sets were collected from the Molecular signatures database (MSigDB) available as part of the GSEA software by restricting the output to the two MSigDB collections C5 (e.g. GO gene sets) and C7 (e.g. immunologic signatures)^34,35^. The significant gene sets were obtained by using p value < 0.01 and FDR < 0.1 as threshold. Ranking of the genes set was done using GSEA v2.0.

Microarrays before and after acute (six hours *in vitro*) and chronic (six months *in vivo*) treatment with GA were also analyzed with the Mode-of-action by Network Analysis (MANTRA) online tool [http:/mantra.tigem.it]^7^. MANTRA’s output is a network where each drug is a node and drugs are connected to each other if they elicit a similar transcriptional response^7^. The ‘distance’ between the connected drugs is a measure of the similarity of their gene expression profiles^8^. The transcriptional distance threshold of ≤0.8 from GA treatment was established to obtain the significant compounds.

### [Ca^2+^]_i_ Measurement

PBMCs of three healthy subjects and of four patients treated with GA, three with INFβ-1a, three with INFβ-1b, and three drug naïve patients, were isolated by Ficoll-Paque density gradients centrifugation.

[Ca^2+^]_i_ was measured by single-cell Fura-2 acetoxymethyl-ester (AM) videoimaging, as previously described^36,37^. PBMCs placed on glass coverslips were first preincubated with anti-CD19 conjugated to fluorescein isothiocyanate (FITC-CD19) for 3 hours to identify B cells. Then, cells were loaded with 10 μmol/L Fura-2AM for 30 minutes at 37 °C in normal Krebs solution containing 5.5 mMKCl, 160 mMNaCl, 1.2 mM MgCl_2_, 1.5 mM CaCl_2_, 10 mM glucose, and 10 mM HEPES-NaOH (pH 7.4). Coverslips were placed into a perfusion chamber (Medical System, Co, Greenvale, NY) mounted on an inverted Zeiss Axiovert 200 microscope (Carl Zeiss) and samples were alternatively illuminated at 340 nm, 380 nm and 490 nm wavelengths. ER Ca^2+^ content was evaluated as cytosolic Ca^2+^ increase elicited by ATP (100 µM) + thapsigargin (1 µM) in a Ca^2+^- free solution. In *ex vivo* experiments, [Ca^2+^]_i_ measurements were conducted 3 hours after plating PBMCs. In *in vitro* experiments, PBMCs were first exposed to 100 µg/ml GA for 8–10 hours, and then analyzed.

### Confocal microscopy

Confocal immunofluorescence procedures in PBMCs were performed as described^38,39^. Cells were fixed in 4% paraformaldehyde in phosphate buffer for 30 minutes. After blocking with 3% BSA, cells were incubated with FITC-CD19 antibodies (1:100; Beckman Coulture) overnight. Nuclei were counterstained with Hoechst (1 μg/ml, Sigma, Milan, Italy). Images were observed using a Zeiss LSM510 META/laser scanning confocal microscope (Carl Zeiss Microscopy, Jena, Germany). Single images were taken with an optical thickness of 0.7 μm and a resolution of 1024 × 1024.

## Data Availability

All data generated or analysed during this study are included in this published article (and its Supplementary Information files).

## References

[1] Weiner HL (2004). Multiple Sclerosis Is an Inflammatory T-Cell–Mediated Autoimmune Disease. Arch. Neurol..

[2] Pröbstel AK, Sanderson NS, Derfuss T (2015). B Cells and Autoantibodies in Multiple Sclerosis. Int. J. Mol. Sci..

[3] Claes N, Fraussen J, Stinissen P, Hupperts R, Somers V (2015). B Cells Are Multifunctional Players in Multiple Sclerosis Pathogenesis: Insights from Therapeutic Interventions. Front. Immunol..

[4] VJohnson KP (2012). Glatiramer acetate for treatment of relapsing-remitting multiple sclerosis. Expert. Rev. Neurother..

[5] Ireland SJ (2014). The effect of glatiramer acetate therapy on functional properties of B cells from patients with relapsing-remitting multiple sclerosis. JAMA Neurol.

[6] Longbrake EE, Cross AH (2016). Effect of Multiple Sclerosis Disease-Modifying Therapies on B Cells and Humoral Immunity. JAMA Neurol.

[7] Iorio F (2010). Discovery of drug mode of action and drug repositioning from transcriptional responses. Proc. Natl. Acad.Sci. USA.

[8] Carrella D (2014). Mantra 2.0: an online collaborative resource for drug mode of action and repurposing by network analysis. Bioinformatics..

[9] Lamb J (2006). The Connectivity Map: using gene-expression signatures to connect small molecules, genes, and disease. Science..

[10] Stettner N (2018). Induction of Nitric-Oxide Metabolism in Enterocytes Alleviates Colitis and Inflammation-Associated Colon Cancer. Cell. Rep..

[11] Wishart DS (2017). DrugBank 5.0: a major update to the DrugBank database for 2018. Nucleic Acids Res.

[12] Gironi M (2008). A pilot trial of low-dose naltrexone in primary progres sive multiple sclerosis. Mult. Scler..

[13] Agrawal YP (2005). Low dose naltrexone therapy in multiple sclerosis. Med. Hypotheses..

[14] Liu X, Singh BB, Ambudkar IS (2003). TRPC1 Is Required for Functional Store-operated Ca Channels. J. Biol. Chem..

[15] Ambudkar IS, Ong HL, Liu X, Bandyopadhyay BC, Cheng KT (2007). TRPC1: The link between functionally distinct store-operated calcium channels. Cell. Calcium..

[16] Lemmon MA, Schlessinger J (2010). Cell signaling by receptor-tyrosine kinases. Cell..

[17] Mori Y (2002). Transient receptor potential 1 regulates capacitative Ca^2+^ entry and Ca^2+^ release from endoplasmic reticulum in B lymphocytes. J. Exp. Med..

[18] Feske S, Prakriya M, Rao A, Lewis RS (2005). A severe defect in CRAC Ca^2+^ channel activation and altered K^+^ channel gating in T cells from immunode icient patients. J. Exp. Med..

[19] Feske S, Skolnik EY, Prakriya M (2012). Ion channels and transporters in lympho cyte function and immunity. Nat. Rev. Immunol..

[20] Feske S, Wulff H, Skolnik EY (2015). Ion channels in innate and adaptive immunity. Annu. Rev. Immunol..

[21] RamaKrishnan AM (2016). Understanding autoimmunity: The ion channel prospective. Autoimmun. Rev..

[22] Starossom SC, Veremeyko T, Dukhinova M, Yung AW, Ponomarev ED (2014). Glatiramer acetate (copaxone) modulates platelet activation and inhibits throm bin-induced calcium influx: possible role of copaxone in targeting platelets during autoimmune neuroinflammation. PLoS One..

[23] Fujiwara T, Oda K, Yokota S, Takatsuki A, Ikehara Y (1988). Brefeldin A causes disassembly of the Golgi complex and accumulation of secretory proteins in the endoplasmic reticulum. J. Biol. Chem..

[24] Ivessa NE, De Lemos-Chiarandini C, Gravotta D, Sabatini DD, Kreibich G (1995). The Brefeldin A-induced retrograde transport from the Golgi apparatus to the endoplasmic reticulum depends on calcium sequestered to intracellular stores. J. Biol. Chem..

[25] Leffler A, Reckzeh J, Nau C (2010). Block of sensory neuronal Na^+^ channels by the secreolytic ambroxol is associated with an interaction with local anesthetic binding sites. Eur. J. Pharmacol..

[26] Beeh KM, Beier J, Esperester A, Paul LD (2008). Antiinflammatory properties of ambroxol. Eur. J. Med. Res..

[27] Yoon C, Giger RJ (2016). Inside Out: Core Network of Transcription Factors Drives Axon Regeneration. Neuron..

[28] Scott LJ, Figgitt DP (2004). Mitoxantrone: a review of its use in multiple sclerosis. CNS Drugs..

[29] Polman CH (2011). Diagnostic criteria for multiple sclerosis: 2010 revisions to the McDonald criteria. Ann. Neurol..

[30] Rostkowska-Nadolska B (2009). Transcriptional activity of genes-encoding kinin B1 and b2 receptors and kinin-dependent genes in nasal polyps. Adv. Med. Sci..

[31] Gautier L, Cope L, Bolstad BM, Irizarry RA (2004). affy-analysis of Affymetrix GeneChip data at the probe level. Bioinformatics..

[32] Baldi P, Long AD (2001). A Bayesian framework for the analysis of microarray expression data: regularized ttest and statistical inferences of gene changes. Bioinformatics..

[33] Edgar R, Domrachev M, Lash AE (2002). Gene expression omnibus: NCBI gene expression and hybridization array data repository. Nucleic Acids Res.

[34] Subramanian A, Tamayo P, Mootha VK (2005). Gene set enrichment analysis: a knowledge-based approach for interpreting genome-wide expression profiles. Proc. Natl. Acad. Sci. USA..

[35] Liberzon A (2015). The Molecular Signatures Database (MSigDB) hallmark gene set collection. Cell. Syst.

[36] Staiano RI (2009). Expression and function of Na^+^/Ca^2+^ exchangers 1 and 3 in human macrophages and monocytes. Eur. J. Immunol..

[37] Secondo A (2015). Involvement of the Na^+^/Ca^2+^ exchanger isoform 1 (NCX1) in neuronal growth factor (NGF)-induced neuronal differentiation through Ca^2+^-dependent Akt phosphorylation. J. Biol. Chem..

[38] Boscia F (2017). The expression and activity of KV3.4 channel subunits are pre cociously upregulated in astrocytes exposed to Aβ oligomers and in astrocytes of Alzheimer's disease Tg2576 mice. Neurobiol. Aging..

[39] Casamassa A (2016). Ncx3 gene ablation impairs oligodendrocyte precursor re sponse and increases susceptibility to experimental autoimmune en cephalomyelitis. Glia..

